# Risk Assessment of Gypsum Amendment on Agricultural Fields: Effects of Sulfate on Riverine Biota

**DOI:** 10.1002/etc.5248

**Published:** 2021-12-29

**Authors:** Krista Rantamo, Hanna Arola, Jukka Aroviita, Heikki Hämälainen, Maija Hannula, Rami Laaksonen, Tiina Laamanen, Matti T. Leppänen, Johanna Salmelin, Jukka T. Syrjänen, Antti Taskinen, Jarno Turunen, Petri Ekholm

**Affiliations:** ^1^ Finnish Environment Institute Jyväskylä/Helsinki/Oulu Finland; ^2^ Department of Environmental and Biological Sciences University of Jyväskylä Jyväskylä Finland; ^3^ Regional Centre of Economic Development, Transport and the Environment of Southwest Finland Turku Finland

**Keywords:** Gypsum, Baltic Sea, sulfate

## Abstract

Gypsum (CaSO_4_∙2H_2_O) amendment is a promising way of decreasing the phosphorus loading of arable lands, and thus preventing aquatic eutrophication. However, in freshwaters with low sulfate concentrations, gypsum‐released sulfate may pose a threat to the biota. To assess such risks, we performed a series of sulfate toxicity tests in the laboratory and conducted field surveys. These field surveys were associated with a large‐scale pilot exercise involving spreading gypsum on agricultural fields covering 18% of the Savijoki River (Finland) catchment area. The gypsum amendment in such fields resulted in approximately a four‐fold increase in the mean sulfate concentration for a 2‐month period, and a transient, early peak reaching approximately 220 mg/L. The sulfate concentration gradually decreased almost to the pregypsum level after 3 years. Laboratory experiments with *Unio crassus* mussels and gypsum‐spiked river water showed significant effects on foot movement activity, which was more intense with the highest sulfate concentration (1100 mg/L) than with the control. Survival of the glochidia after 24 and 48 h of exposure was not significantly affected by sulfate concentrations up to 1000 mg/L, nor was the length growth of the moss *Fontinalis antipyretica* affected. The field studies on benthic algal biomass accrual, mussel and fish density, and *Salmo trutta* embryo survival did not show gypsum amendment effects. Gypsum treatment did not raise the sulfate concentrations even to a level just close to critical for the biota studied. However, because the effects of sulfate are dependent on both the spatial and the temporal contexts, we advocate water quality and biota monitoring with proper temporal and spatial control in rivers within gypsum treatment areas. *Environ Toxicol Chem* 2022;41:108–121. © 2021 The Authors. *Environmental Toxicology and Chemistry* published by Wiley Periodicals LLC on behalf of SETAC.

## INTRODUCTION

Aquatic eutrophication caused by agricultural nutrient loading is an environmental problem projected to become even more severe in the future due to the increasing population, the changes in people's diet, and climate change (Tilman et al., 2001). Gypsum amendment is a promising, cost‐effective, and socially acceptable way to cut down the phosphorus loading of coastal waters from large agricultural areas (Ekholm et al., 2012; Ollikainen et al., 2020). Gypsum (CaSO_4_∙2H_2_O) applied on the surfaces of fields gradually dissolves in the soil. The divalent calcium and sulfate ions effectively increase the ionic strength of the soil solution, which enhances the aggregation of soil particles, making them less prone to erosion. The phosphorus losses are reduced not only by the lower erosion and lesser transport of the phosphorus bound to the soil particles but also by the reduced desorption of phosphorus from the soil particles (Uusitalo et al., 2012). According to the evidence gathered so far, gypsum can halve the phosphorus losses from amended fields, and the effect of this can be nearly immediate and can last for at least 3 years (Ekholm et al., 2012; Uusitalo et al., 2012). Whether gypsum induces more permanent beneficial changes in the soil structure, however, is not yet known. Gypsum is a relatively soluble compound, and because sulfate and calcium are weakly bound to the soil, they are gradually flushed away from the soil via surface runoff and drainage flow. This increases the concentrations of sulfate and calcium in the downstream waters. Between these two ions, sulfate in high concentrations has been reported to have harmful effects on freshwater biota (Elphick et al., 2011). Hence, gypsum amendment may pose an ecological risk, which must be assessed before the method is widely applied.

Sulfate concentrations are naturally high in seawater and in inland waters, especially in arid areas. In such environments, the limited sulfate loading from gypsum amendment hardly affects the concentrations or threatens the biota. However, most freshwaters in boreal regions have a naturally low sulfate content (Ekholm et al., 2020) and may thus be more sensitive to elevated sulfate loading. In lentic waters, sulfate may accelerate eutrophication by increasing the benthic flux of phosphorus (Smolders & Roelofs, 1993). In cases of massive loading, sulfate may also cause saltation of hypolimnion and salinity stratification, which may further increase the release of phosphorus from sediments. Therefore, gypsum amendment should not be applied to fields within lake catchments. No such problems are foreseen in running waters, but the elevated sulfate concentrations caused by gypsum treatment may be directly harmful to biota.

Protective concentrations have been calculated and proposed by Elphick et al. (2011) and Sahlin and Ågerstrand (2018), but quality standards for surface waters have been set only in British Columbia, Canada (Meays & Nordin, 2013) and Illinois, United States (Illinois Pollution Control Board, 2011). Risk assessment of anionic sulfate is not a straightforward task because the toxicity test results are dependent on the test conditions and water quality and are thus weakly transferable to field conditions. Several studies (see Elphick et al., 2011) have shown that sulfate becomes less toxic with increased water hardness and particularly with increasing calcium concentration. The mechanism is likely related to either the competition between the ions at the binding sites on the respiratory epithelium or the change in the degree of gill membrane permeability (Elphick et al., 2011). Hence, the concomitantly increased losses of calcium from the gypsum‐treated fields may counteract the potential harmful effects of increased sulfate. However, we do not know whether these interactions are similar at our field sites, where the sodium, calcium, and sulfate concentrations differ from those in the literature on sodium sulfate. Other interactions are also possible, because the expected decreasing concentrations of nutrients and suspended solids in the recipient streams can have positive ecological effects.

In 2016, a large‐scale pilot study was started in southwestern Finland to investigate the feasibility and effects of gypsum amendment in a real‐world setting. Gypsum was spread on 15 km^2^ of clayey agricultural fields at the Savijoki River catchment, and the effect of such on phosphorus loss was intensively monitored. The major goal of our study was to determine whether gypsum amendment could be used to reduce phosphorus transport to streams and rivers, and finally to the Baltic Sea, which is suffering from severe eutrophication (Helsinki Commission [HELCOM], 2018). The sulfate concentrations in the rivers resulting from the gypsum treatment and the toxicity of these concentrations to the biota in the recipient streams could not be reliably anticipated from the existing knowledge. Therefore, there was a need for an ecological risk assessment combining exposure and its effects to ensure the environmental safety of the treatment before its wider application. In the present study, we performed an extensive assessment of the risks posed by gypsum treatment to the riverine biota within the Savijoki River catchment. We combined the results of several controlled laboratory assays, in situ exposures, and field surveys. The target organism covered a range of taxa at different trophic levels and included species of both ecological and conservational importance.

## MATERIALS AND METHODS

### Study area

**Figure 1:**
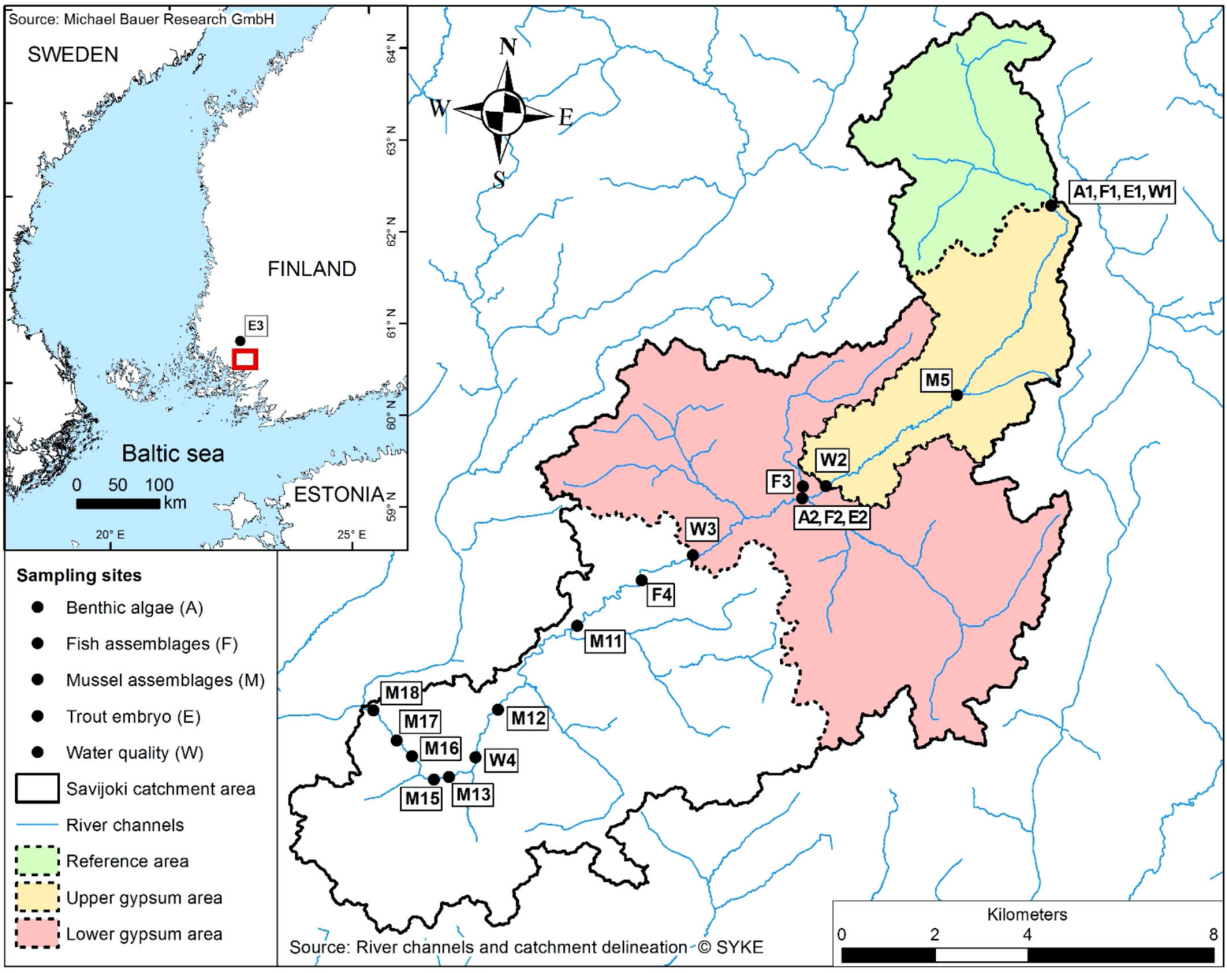
The study area, reference and gypsum‐amended subcatchments, and field sampling sites in the Savijoki River catchment, and its location in Finland (inset).

The pilot area included the upper and central reaches of the Savijoki River, a tributary of the Aurajoki River, which discharges into the Archipelago Sea (the Baltic Sea) in southwestern Finland (Figure 1). The catchment of the Archipelago Sea is defined as an agricultural hotspot by HELCOM owing to the intensive agriculture practiced on fine‐textured erodible soils, the erosion clearly seen in the highly turbid river waters. The ecological status of the Savijoki River has been categorized as poor (Finland's Environmental Administration, 2020). Much of the nutrient and suspended‐solid loads entering the river originates from agricultural fields, which account for up to 50% of the catchment area (Table 1). The agriculture in the fields is dominated by cereal farming, with the number of livestock being low. Most of the field soils are classified as Vertic cambisoil, and the minority consists of coarser soils. The remaining areas are mainly forest, with a small share of constructed areas. Except for the sparse human settlements, there are no other known loading sources. There are no lakes in the catchment, and the fields are efficiently drained. Therefore, the runoff is close to 0 in the dry periods in summer and winter but may exceed 200 L/s/km^2^ due to the spring snowmelt and autumn rains.

**Table 1:** Sub‐basins and characteristics of gypsum treatment and reference areas in the Savijoki

Sub‐basin	Area (km^2^)	Field (%)	Gypsum‐amended fields of all fields (%)	Forest (%)	Constructed (%)
Reference	15.0	38.7	0	56.2	3.9
Upper gypsum	17.7	49.9	46.7	42.3	7.6
Lower gypsum	49.1	42.0	52.5	51.6	4.7

The upper reaches of the river were left as a reference area where no gypsum was used whereas approximately half of the fields in the central reaches were amended with gypsum in August–October 2016. A total of 6000 tons of gypsum was spread on 14.9 km^2^ of agricultural fields, translating to 2780 tons of sulfate. Water samples were taken from three sites in the Savijoki River representing the reference area (“Mittapato,” W1 in Figure 1), from the outlet of the upper gypsum area (“Yliskulma,” W2), and from the outlet of the lower gypsum area (“Parmaharju,” W3). The sampling frequency exceeded 20 times a year, and the samples were analyzed for sulfate concentrations (ion chromatographic determination), among others. In addition, a station chosen to represent high abundance of the threatened mussel *Unio crassus* in the lower reaches (W4) was monitored 34 times in the first 2 years after the amendment. To complement the manual water sampling, online sensors that recorded the turbidity and electric conductivity at hourly intervals, among others, were deployed at the three uppermost sites. A nonlinear mixed effects model was made for the relationship between the hourly electric conductivity observations given by the sensors and the sulfate concentrations determined from the manually taken samples (see the Supporting Information). The use of a power relationship is theoretically justified by the increase in divalent calcium and sulfate ions at the sites and in periods during which gypsum had an effect on the electric conductivity. Other anions and cations were also measured from grab samples before and after the amendment. The runoff was measured at the outlet of the reference area using a calibrated V‐notch weir (Supporting Information, Figure S1).

The same type of gypsum (originating from the Yara Siilinjärvi, Finland, plant) that was used in the pilot study was also used in the risk assessment experiments. The gypsum is a byproduct of the manufacture of phosphoric acid from apatite mineral. The water that was used in the laboratory experiments was from the Savijoki River reference area.

### Test organisms

The toxicity of sulfate was assessed for several organisms at different trophic levels in the laboratory experiments (the moss *Fontinalis antipyretica* Hedw., the unionid clam *U. crassus* Philipsson), through in situ assays (the eggs of sea‐migrating brown trout *Salmo trutta* Linnaeus and benthic algae), and by field surveys (mussels and fish).

#### Primary producers

The greater water‐moss *F. antipyretica* is a bryophyte typically abundant in Finnish streams (Rääpysjärvi et al., 2016), including the Savijoki and other rivers in the areas forming potential target catchments for gypsum treatment. Aquatic mosses form key microhabitats in boreal streams (Stream Bryophyte Group, 1999) and are important in stream ecosystem functioning. However, mosses have a low growth rate (Furness & Grime, 1982) and therefore low resilience for disturbances. Hence, the deterioration of mosses can lead to a cascade of harmful effects in boreal‐stream ecosystems (Turunen et al., 2020). In previous laboratory studies, sulfate at high concentrations has reduced the growth and chlorophyll content of *F. antipyretica* (Davies, 2006; Elphick et al., 2011). Benthic algae (periphyton) also form a key foundation of stream food webs, and their abundance is dependent on the availability of light and dissolved nutrients. Gypsum treatment can increase algal growth (by decreasing the water turbidity) or decrease it (by decreasing the phosphorus concentrations). There have been no studies on the sulfate effects on benthic algae, but the growth of the pelagic species *Raphidocellis subcapitata* was reported to have been reduced in concentrations exceeding 1000 mg/L (Elphick et al., 2011).

#### Mussels

The thick‐shelled river mussel *U. crassus* is classified as an endangered and protected species (Hyvärinen et al., 2019) living in the rivers of the study area. Some other mussel species have been used in sulfate toxicity experiments (see Wang et al., 2016), but to the best of our knowledge, *U. crassus* has not been used for such purpose. Excessive concentrations of suspended inorganic solids in water can impede the food intake and respiration of mussels (Tuttle‐Raycraft et al., 2017). Therefore, if gypsum treatment indeed decreases the inorganic turbidity of the runoff from the fields, this can even be beneficial for the mussels if sulfate would not pose a risk to them.

#### Fish

The migratory brown trout *S. trutta* is an endangered species in the Baltic Sea region in Finland (Hyvärinen et al., 2019). There are tens of potential trout streams along the south and southwest coast of Finland where gypsum amendment can be applied. Wild trout parr or resident individuals have been occasionally observed in the Savijoki River, and there is some indication of natural reproduction in its tributaries (Koski et al., 2013). Elevated sulfate concentrations, however, can be toxic to the early life stages of salmonids (Elphick et al., 2011). On the other hand, gypsum treatment can reduce the phosphorus concentration and turbidity, alleviating the eutrophication‐related oxygen demand and sedimentation. This can even increase the survival of embryos.

### Laboratory studies

#### *Growth of the moss* F. antipyretica

We conducted a laboratory experiment to test the effect of gypsum on the length and biomass growth of *F. antipyretica*. The mosses that were used in the experiment were collected from the unpolluted Muuramenjoki River (62°7.8′N, 25°39.9′E, Central Finland) and were acclimatized for 3 days to the test conditions (the Savijoki water with aeration at 18 °C room temperature and in a 16:8‐h light:dark cycle). A part of the collected moss was retained in the water from the Muuramenjoki, which served as the second control for the experiment. The waters were stored at 5°C for 3 days before the experiments. The gypsum was dried at 60°C overnight and was then mixed in with the test waters that had been sieved with a 38‐µm mesh to remove the excess particles. The natural sulfate concentrations in the control waters of the Muuramenjoki and the Savijoki, respectively, were 3.9 and 13 mg/L. The nominal test concentrations were 30, 200 (the expected minimum and maximum concentrations in the Savijoki after the gypsum amendment, respectively), 400, 600, and 1200 mg/L (saturated gypsum solution). The measured concentrations were 42, 220, 453, 650, and 1200 mg/L and were based on the samples pooled from the replicates. Each concentration was tested in 100‐ml glass jars in 10 replicates.

The test broadly followed the protocol described by Davies (2006). Two‐centimeter‐long apical segments of *F. antipyretica* were cut and kept in the Savijoki water during the test preparation. Three randomly taken shoots were then tapped on paper tissue to remove the surficial water, and their combined fresh mass was taken with a microbalance before they were placed in the test vials. The 21‐day experiment was conducted at a constant temperature of 18°C and in a 16:8‐h light:dark cycle. The test waters were changed at 3‐day intervals. Water samples were taken on days 0, 8, and 16 for sulfate analysis. Sulfate was analyzed in the laboratory of the Finnish Environment Institute according to the ISO 10304‐1:2007 standard, using ion chromatography. The oxygen concentration, pH, conductivity, and temperature were measured at 3‐day intervals (using a WTW Multi 3430 multiparameter meter). At the end of the experiment, the lengths and the fresh weights of the shoots were determined again. For measuring the dry mass, we dried the shoots overnight in an oven at 105°C, in aluminum cups.

#### *Survival of the mussel* U. crassus *glochidia*

We tested the effects of sulfate from gypsum on the survival of the glochidia larvae of *U. crassus* in acute 24‐ and 48‐h static exposure, applying the ASTM International (2017) guidelines as close as possible to this endangered species. Thirty gravid mussels were collected from the Perniönjoki watershed, Yliskylä, Finland, situated in the Kiskonjoki catchment at 60°16.5′N and 23°7.8′E, on May 17, 2017, and were transported in cooled boxes in river water, with constant aeration, to the laboratory, where the mussels were slowly acclimatized to the 15°C test water temperature. To allow glochidia development, the adult mussels were maintained in the laboratory for 3 weeks, with constant aeration. They were fed with green algae (*R. subcapitata*), and the water was partially changed three times a week. After 3 weeks, all the mussels were alive, and we returned them to their native river.

At the onset of the testing, the containers were searched daily, and the glochidia were collected where the gravid mussels had released them. Their viability was tested with saturated NaCl solution. The viability was over 90%. The glochidia that were used in the tests were less than 24 h old. The test water transported from the Savijoki and the water from Perniönjoki served as additional controls and were stored at 5°C before the experiments. The gypsum was dried at 60°C overnight and was mixed in with the test waters that had been sieved with a 38‐µm mesh to remove the excess particles. The toxicity testing included eight treatments with four (24‐h test) or three (48‐h test) replicates each at 15 ± 1°C in a 20:4‐h light:dark cycle. The natural sulfate concentrations in the Savijoki and the Perniönjoki control waters were 19 and 10 mg/L, respectively. The nominal test concentrations of 40, 60, 120, 250, 500, and 1000 mg/L SO_4_ (average measured concentrations of 38, 66, 120, 247, 493, and 957 mg SO_4_/L and 40, 66, 130, 263, 520, and 1017 mg/L SO_4_ in the 24‐ and 48‐h tests, respectively) were used. We placed 15–20 glochidia into each glass vial with 20 ml of test water using a glass Pasteur pipette, and covered the vials with parafilm. The exact total number of glochidia/treatment level, as counted after the exposures, was 74–81 in the 24‐h test and 41–61 in the 48‐h test. Water samples were taken three times (before, during, and at the end of the experiment) for sulfate concentration analyses. We measured the oxygen concentration, pH, conductivity, and temperature at 3‐day intervals.

The survival (%) was examined using a dissecting microscope, by pipetting a drop of saturated NaCl solution next to the glochidia in a Petri dish with a black background. The individuals whose valves were closed before NaCl addition and that did not respond to our gentle knocking on their shells were considered dead. Those that snapped shut their open shells in response to NaCl addition were considered alive, and those that remained open were counted as dead.

#### *Behavior of the adult* U. crassus *mussels*

We studied the effects of sulfate on the behavior of adult *U. crassus* during 4‐day sulfate exposure. Thirty‐six mussels were collected from the Perniönjoki on October 6, 2016. The mussels were transported to the laboratory in insulated boxes with aeration, and were acclimatized to the test room conditions (18°C; 16:8‐h light:dark cycle; the Savijoki water) for 3 days. At the end of the first day, the mussels were fed with green algae (*R. subcapitata*). The exposure was conducted in the Savijoki water, in 2‐L beakers with aeration for 4 days in the test room. The 13‐mg/L natural sulfate concentration of the Savijoki served as a control for the experiment. The nominal test concentrations were 30, 200, and 1200 mg/L SO_4_, and the measured concentrations were 43, 210, and 1100 mg/L SO_4_ on average. Eight mussels were randomly chosen for each treatment and were individually held in the aerated exposure beakers. In addition, three beakers containing only the Savijoki water served as a negative control for the food consumption measurements. The test waters were changed once (after 2 days) during the 4‐day experiment. We fed each mussel with the same amount of *R. subcapitata* after the water change and at the end of the experiment.

The behavior of the mussels was monitored five times every day: at 8 a.m., 10 a.m., 12 p.m., 2 p.m., and 4 p.m. Three different types of behavior were recorded: opening of the shell (open/closed), filtering activity (siphons in/out), and foot movement (foot out/in). The mussels gained 1 point/mode of activity. In uncertain cases, 0.5 point was given. For each individual, the points for every mode of activity and for all modes of activity were summed up and were then averaged within a treatment group. At the end of the experiment, we checked the survival of the mussels by knocking gently on the shell. The individuals that did not react by closing their shells were considered dead.

To study the food consumption of the mussels, we took water samples from the beakers right after and 2 h after the last feeding, for algal cell counting. The samples were preserved with Lugol's solution and stored at 5°C for microscope counting. The samples were first homogenized with a vortex mixer, and the number of algal cells in 1 ml water was then determined using a Bürker chamber and a microscope. We calculated the food consumption of each mussel as the difference between the initial cell concentration and the final cell concentration after 2 h.

Water samples were taken from the test waters three times (before, during, and at the end of the experiment) for sulfate concentration analysis. We also monitored the oxygen concentration, pH, conductivity, and temperature thrice during the experiment, simultaneously with water sampling.

After the 4‐day exposure and visual monitoring, the mussels' behavioral responses were measured with a Multispecies Freshwater Biomonitor (MFB®) device (Gerhardt et al., 1998). The MFB quantifies movements, which induce changes in the weak electric field in the test chambers and reveal the percentages of time that the animal spends in certain activity modes, characterized by different frequency classes up to 8.5 Hz. One mussel was placed in each test chamber, with eight replicates/four treatments, and the mussel's behavior was measured for 2 h at 10‐min intervals. Eight mussels were set aside for the preliminary tests, in which the behavior types were visually observed simultaneously with the MFB oscilloscope function to define the behavioral responses: inactivity, foot movement, filtering, and valve opening/closing. The signal amplitudes of these behavior types differed, but the frequencies overlapped, ranging from 0.5 to 2.5 Hz. Thus, for the statistical analyses, the overall activity in the less than 2.5‐Hz range was calculated. After the experiments, we kept the mussels in clean Savijoki water for 1 month to allow them to recover, and then we returned them to their home river.

### Field studies

#### Benthic algal accrual

We studied the benthic algal biomass accrual in the Savijoki before the gypsum amendment (Autumn 2016) and after the gypsum amendment (2017), at a site downstream of the gypsum spread area (lower gypsum area, A2 in Figure 1), and at an upstream reference site (reference site, A1). In both years and sites, we placed 6–10 unglazed 5 × 5‐cm clay tiles into a riffle habitat for 7 weeks. After the incubation period, we estimated the biomass of algae on each tile by the mass of chlorophyll a using two methods: in the field from the tiles using the portable fluorometric probe BenthoTorch^TM^ (bbe Moldaenke; Harris & Graham, 2015) and in the laboratory of the Finnish Environment Institute in Oulu via spectrophotometry of the samples obtained from tiles frozen in the field.

#### Mussel assemblages

The mussel abundance was initially surveyed on September 3–23, 2016, while the gypsum was spreading in 18 sites along the Savijoki. Even though the gypsum spread had started at the beginning of August, we considered that the aforementioned survey period represented a reference period for the treatment due to the very dry weather, low runoff, and limited sulfate concentration increase in the Savijoki water during such a period (Supporting Information, Figure S1). The reaches within the reference and gypsum spread areas, however, were generally not favorable for mussel populations. Thus we selected eight sites mostly from the lower reaches of the river for the study (Figure 1). Fixed transects of known length and width across the river channel (sites M15–18) or along the river channel (sites M5, M11–13) were established for species identification and counting. The shallow‐ and deep‐water transects were surveyed by wading and diving, respectively, and all the mussels within the transects were collected, the species they belonged to were identified, and they were measured, counted, and then returned to their original habitat. This was done once before the gypsum treatment (in 2016) and twice after the gypsum treatment (in 2017 and 2018).

#### Fish assemblages

To assess the impact of gypsum amendment on stream fish assemblages, we conducted electrofishing at four sites in the Savijoki in Autumn 2017. Three sites (Figure 1) were in the gypsum spread area (F2, F3 tributary site, and F4), and one site (F1) was the reference site above the gypsum treatment area. In addition, we had electrofishing results from previous autumns from F2 (2013), F3 (2012), and F4 (2012), which we used to compare the fish densities before and after the gypsum amendment.

In each site, we fished in a riffle habitat with a backpack electrofishing device (Hans Grassl IG20002C30) using the single‐pass removal method, and we measured the sampling area. We identified the species to which the captured fish belonged, measured their lengths, counted them to estimate the densities, and then released them back to the river. The pure catch was used in estimating the density; no catchability value was used.

#### *Survival and development of* S. trutta *embryos*

The trout eggs were incubated in situ at three sites after the gypsum treatment. In the Savijoki, a test site (E2 in Figure 1) and a reference site (E1) were situated downstream and upstream of the gypsum treatment area, respectively. An additional reference site (E3), which represented a woodland catchment, was near the Järvijoki River, another tributary of the Aurajoki River.

Eggs of the sea‐migrating brown trout of the Isojoki River stock, managed by the Natural Resources Institute Finland in the Laukaa hatchery, were fertilized in the hatchery on October 25, 2017. The following day, the eggs were disinfected with Buffodine® and were placed in 0.5‐L plastic bottles (50 eggs in each) filled with water. On the same day, the bottles were transported on ice to the study sites, where 50 eggs were poured into each incubation cylinder. The cylinder, baskets, gravel, and the exact method that was used were obtained from Harris (1973) and Syrjänen et al. (2008). Each basket contained four cylinders, and three baskets were placed at each of the three study sites. The baskets were slightly buried into the bottom and supported with gravel and stones. The distances between the baskets were within 0.8–7.0 m at each site.

The exact incubation sites were selected based on the knowledge of the microhabitat conditions in real trout redds (see Syrjänen et al., 2014). The water depth and current velocity around each basket were measured as microhabitat descriptors at each field visit (Supporting Information, Tables S2 and S3). The accumulation of fine particles in each cylinder was visually evaluated on a tray in the field. The maximum size of the particles was assumed to be the same as the cylinder mesh size (2 mm). At the end of the experiment, one cylinder and one basket from each site were collected for a detailed particle analysis at the University of Jyväskylä (Supporting Information, Table S4). According to the dried particles' maximum diameters, they were categorized into fine particles (0–8 mm) and coarser particles (8–16, 16–32, 32–64, 64–128, and 128–256 mm) and were weighed. The proportion of less than 2‐mm fraction was measured by sieving the 0–8‐mm fraction through 0.5, 1.0, and 2.0‐mm sieves.

A water temperature logger (HOBO® Pendant®Temp/Light, 8 K, 1‐800‐loggers, OnSet, 0.2°C accuracy) was placed in one of the baskets at each site. The loggers measured the water temperature four times a day, and the degree days were calculated according to the daily mean temperature (Supporting Information, Table S4).

Water quality data from the incubation sites were obtained from the Water Quality Database of the Finnish Environment Institute (2018a). The Savijoki water quality was monitored during the incubation period at three sites, one near the reference site and two near the gypsum site (Supporting Information, Table S5). For Järvijoki, water quality data of the downstream area from the incubation site and of the upstream Lake Savojärvi for the years 2000–2016 were obtained from the Water Quality Database (Finnish Environment Institute, 2018b; Supporting Information, Table S6).

The incubation conditions were inspected on January 12, 2018. Sand had accumulated into the E3 incubation baskets, and bottom ice was observed in the E2 site. Egg samples were collected on four occasions: on March 25, April 19, May 8, and May 21, 2018. However, in March, eggs could not be sampled from the Savijoki sites due to several overlapping ice layers. At each sampling occasion, one cylinder was taken from each basket. The living and dead embryos and the hatched alevins were counted on a tray, and one to five living individuals from each cylinder were collected for length measurements. The total length of the embryos was measured in the laboratory of the University of Jyväskylä the following day. The hatched alevins were photographed in the field.

### Statistical methods

To control for the dependence of the absolute mass increment of *F. antipyretica* on the initial mass at the start of the experiment, the proportional increment of fresh mass (FM) was calculated as (final FM − initial FM)/initial FM. This and other growth variables of the treatment groups were compared using analysis of variance (ANOVA), followed by Tukey's post hoc test to localize the significant differences. However, we also used Dunnett's *t*‐test to specifically evaluate the differences between the test concentrations and the control.

The proportion of survived glochidia and the data from both the visual and MFB measurements of the behaviors of the adult mussels were not normally distributed even after arcsin transformations, according to the Shapiro–Wilk test. The nonparametric Kruskal–Wallis test was thus used to test the differences among the treatments (*α* = 0.05; two‐sided test). If significant differences were found, pairwise comparisons with Bonferroni correction were performed. The differences in the food consumption of the mussels among the treatment levels were tested using one‐way ANOVA and Tukey's post hoc test.

The effect of the gypsum amendment on the benthic algae accrual (logarithm‐transformed chlorophyll a concentrations) was tested with two‐way ANOVA, using time (before/after) and site (reference/impact) as factors, where the interaction (site × year) indicates the effect of the gypsum treatment.

Because we did not have a reference site in the mussel survey, the abundance of *U. crassus* and the total mussel abundance were compared only by study year, using repeated‐measures ANOVA.

The estimated total fish densities and the densities of the most common species before and after gypsum treatment were compared with paired *t*‐tests. The F1 reference had no previous fishing data and was used only for the contemporary comparison of the catches after gypsum treatment. The trout egg survival rate (%) for each cylinder was calculated as the proportion of living embryos in relation to the original number of eggs (50 eggs). Arcsin transformation was used for this purpose. The proportion of living embryos between the sites and the field rounds was tested with two‐way ANOVA. The length is given as the mean body length of the measured individuals in each cylinder.

## RESULTS AND DISCUSSION

Gypsum amendment increased the sulfate concentration in the Savijoki from a level of less than 20 mg/L to a short‐lived maximum of approximately 220 mg/L (Supporting Information, Figure S3). The highest seasonal mean concentration (60.3 at the upper gypsum area and 75.7 mg/L at the lower gypsum area) occurred during the 2‐month period immediately after the gypsum amendment (Table 2), after which the mean concentrations gradually decreased. Three years after the gypsum amendment, the sulfate concentrations in the gypsum area were at approximately pregypsum level. The concentrations of other ions also increased (Supporting Information, Figure S4) partly due to the cation exchange reactions triggered by the calcium in the soil (Uusitalo et al., 2012). The mean turbidity levels and the concentrations of dissolved reactive phosphorus were strongly affected by the seasonally changing runoff, which largely masked the relatively modest effect of gypsum on the arithmetic mean values (Table 2).

**Table 2:** Mean runoff at reference site (W1 in Figure 1), sulfate concentrations, turbidity, and dissolved reactive phosphorus concentrations in reference, upper gypsum (W2), and lower gypsum (W3) sites in the Savijoki River by periods relative to gypsum amendment

			Sulfate (mg/L)			Turbidity (NTU)			Dissolved reactive P (μg/L)		
Period		Runoff (L/s/km^2^)	Reference	Upper gypsum	Lower gypsum	Reference	Upper gypsum	Lower gypsum	Reference	Upper gypsum	Lower gypsum
Before	19/2–31/7/16	7.9	10.9	16.8	21.0	69	58	58	21	25	21
During	1/8–31/10/16	0.1	16.0	34.4	49.7	44	30	30	20	17	15
After gypsum	1/11–31/12/16	2.3	16.2	60.3	75.7	62	59	48	21	14	15
	1/1–30/6/17	4.7	12.3	32.6	42.7	55	59	53	30	29	30
	1/7–31/12/17	12.7	10.7	28.3	39.1	104	105	95	40	44	33
	1/1–30/6/18	9.1	12.3	19.8	25.6	63	58	57	33	47	48
	1/7–31/12/18	2.0	17.6	28.4	36.8	28	36	42	24	21	18
	1/1–15/5/19	16.7	9.5	16.7	21.1	51	56	56	27	33	29
	20/9–31/12/19	25.4	8.1	14.1	19.0	139	156	154	40	52	45

NTU = nephelometric turbidity unit.

Critical analyses for estimating the protective sulfate concentrations in freshwaters are scarce, and quality standards are rarely applied in different jurisdictions. Elphick et al. (2011) separately derived the sulfate concentrations for soft water conditions (10–40 mg/L CaCO_3_) and moderately hard water conditions (80–100 mg/L CaCO_3_) using sublethal responses. The species sensitivity distribution approach yielded 129 and 644 mg/L SO_4_, respectively, and the assessment factor approach yielded 75 and 625 mg/L SO_4_. Sahlin and Ågerstrand (2018) derived protective long‐term annual average and short‐term maximum acceptable environmental quality standard (AA‐EQS and MAC‐EQS) proposals applying European Communities technical guidance (European Commission, 2011). The protective AA‐EQS sulfate concentrations ranged from 15 to 42 mg/L SO_4_ for 40–100 mg/L CaCO_3_ hardness, and the protective MAC‐EQS sulfate concentrations ranged from 96 to 158 mg/L SO_4_, both derived using the assessment factor approach. Gypsum treatment increased the calcium concentration, and the average hardness for 2 years after the amendment was 85 mg/L CaCO_3_ at the outflow of the upper gypsum area (W2) and 94 mg/L CaCO_3_ at the outflow of the lower gypsum area (W3). Hence, a comparison of the measured sulfate concentrations in the river (Table 2) with the quality standard estimates by Elphick et al. (2011) would suggest no risks, whereas a comparison with the quality standard estimates by Sahlin and Ågerstrand (2018) would suggest that the most sensitive species may have experienced adverse effects. The data sets in these two studies reveal a common problem in the derivation of quality standards: the lack of data and thus uncertainty in the extrapolation (Korkaric et al., 2019). Sulfate is a substance whose risk assessment requires more ecotoxicity data, especially in varying water chemistries. Thus our study attempted to go beyond a simple environmental‐quality standard versus measured‐concentrations comparison and applied the weight‐of‐evidence approach using local species in the laboratory and field settings.

### Growth of moss

**Figure 2:**
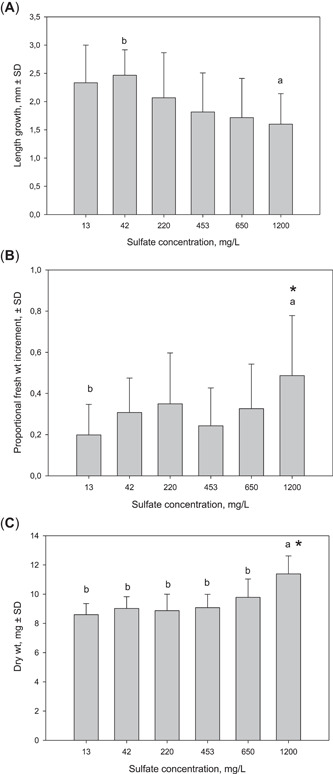
The average (±SE; *n* = 10) of (**A**) length growth, (**B**) proportional fresh mass increment (mass in the end—mass at the start/mass at the start), and (**C**) final dry masses of *Fontinalis antipyretica* in different sulfate concentrations. The statistically significant differences. among the treatments (analysis of variance and Tukey's test) and from the control (13 mg/L, Dunnett test) are marked with the symbols a and b or with an asterisk, respectively.

All the measured growth variables (length growth, proportional fresh mass increment, and dry mass) differed among the treatment groups. The length growth declined with increasing sulfate concentration (Figure 2). However, the Dunnett test suggested that only at the highest concentration did the length growth differ from the control (*p* = 0.059, marginally significant at the standard *α* level), being approximately 31% smaller on average. The Tukey test showed a significant (*p* = 0.047) difference only between the highest and the lowest spiked test concentration (42 mg/L).

In nature, many seasonally varying factors (e.g., depth, light availability, nutrients, thermal conditions) affect the growth of aquatic mosses (Furness & Grime, 1982). However, assuming that the growth rate estimated in our experiments will be constant and independent of size, *F. antipyretica* will grow 41 mm/year on average with the pregypsum sulfate concentration (13 mg/L) of the Savijoki, and 13 mm (31%) less with the highest test concentration (1200 mg/L). With the test concentration (453 mg/L) closest to the short‐lived sulfate peak observed in the Savijoki (220 mg/L), the annual growth will be 9 mm (22%) less than with the natural sulfate concentration. However, such high sulfate peaks were only sporadic, and because gypsum amendment was applied in fall, outside the main growing season, these peaks will probably not significantly affect the length growth of mosses, if at all.

In contrast to length, mass growth seemed to increase with the sulfate concentration. The proportional fresh mass increment (*p* = 0.02) and the dry mass (*p* < 0.001) were greater with the highest sulfate concentration than with the control (Figure 2). The dry mass with the highest sulfate concentration also differed from that with all the other treatment levels (*p* ≤ 0.014). Some precipitation of solids on the moss leaves was visually witnessed during the experiment. This attached material, which could not be removed without damaging the moss, might have been either gypsum or calcium carbonate. In addition to causing the increase in mass, it might have hindered the length growth. For further studies, we would recommend using carbon sequestration as the parameter for measuring *F. antipyretica* growth (Sand‐Jensen & Madsen, 1991).

The sulfate toxicity results reported by published papers are not highly comparable due to the differences in test conditions, such as the water hardness and the sulfate compounds used (Davies, 2006; Elphick et al., 2011). However, Davies (2006) and Elphick et al. (2011) have previously reported results similar to ours: that the length growth of *F. antipyretica* decreased with high sulfate concentrations. The 1200 mg/L apparent sulfate concentration effect was lower than the median effect concentration (EC50) value (greater than 2522 mg/L) estimated by Elphick et al. (2011) for the growth of *F. antipyretica*. However, the broad estimate of the no‐observed‐effect concentration (NOEC) value from our study would be 650 mg/L sulfate, which is within the 603–654 mg/L range estimated by Elphick et al. (2011). Even though the NOEC values have been under debate (see Green et al., 2012), the existing data from short‐term experiments suggest that sulfate is harmful to *F. antipyretica* only at very high concentrations. However, long‐term effects of sulfate may appear even at lower concentrations, and studying these is warranted.

### Survival of mussel glochidia

The survival rates of the control glochidia were on average 87.1% (±2.5%; Perniönjoki) and 92.6% (±4.9%; Savijoki) after 24 h of exposure and 86.1% (±13.4%) and 88.6% (±6.9%) after 48 h of exposure. There were no differences in the proportion of survived glochidia among the tested sulfate concentrations after 24 h of exposure (proportion range: 0.75–1.00; *χ* ^2^ = 7.295; *df* = 7; *p* = 0.399) and after 48 h of exposure (range: 0.56–1.00; *χ* ^2^ = 6.368; *df* = 7; *p* = 0.498; Supporting Information, Figure S4). This critical stage of the *U. crassus* life cycle seems not to be threatened by gypsum amendment. The valve closing is an ecologically relevant endpoint because it indicates the infectivity of glochidia, that is, their ability to attach to fish gills or fins, which allows their development into juveniles (Fritts et al., 2014).

The aforementioned results are in line with those of previous experiments with invertebrates. Calcium sulfate was not acutely toxic below the saturation concentrations to the crustacean water flea *Ceriodaphnia dubia* (Mount et al., 2016). The estimated effect concentration (96‐h EC50) of sodium sulfate (Na_2_SO_4_) varied from 1338 to 2709 mg/L SO_4_ among the juveniles of five bivalvian species (Wang et al., 2017), which is higher than the maximum concentration in our study (1020 mg/L SO_4_). Gypsum amendment in fall should also reduce the potential exposure because in this region, *U. crassus* release their glochidia in spring and early summer, when the runoff and sulfate losses from the fields are also low.

### Behavior of adult mussels

**Figure 3:**
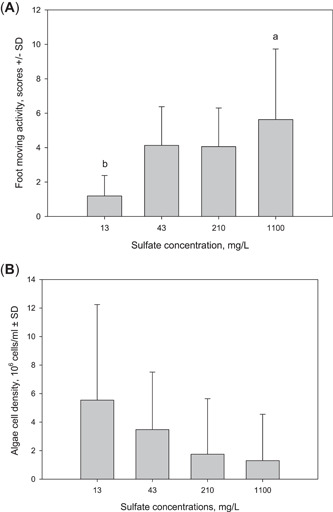
The average (±SD; *n* = 8) of (**A**) foot movement activity of *Unio crassus* and (**B**) decrease in algal concentration in different exposure concentrations of sulfate. The statistically significant differences between the treatment groups are indicated with the letters a and b.

All the mussels were alive at the end of the exposure. The foot movement activity increased with increasing sulfate concentration and was six times as high as the control value (*p* = 0.021; Figure 3A) at the highest concentration. The other activity types did not show differences among the treatment levels. However, the consumption of algae seemed to have decreased with increasing sulfate concentration (Figure 3B), even though there were no statistical differences between the treatment levels. The behavioral activity of the adult mussels differed between the treatments (*χ* ^2^ = 13.980; *df* = 3; *p* = 0.003) in the MFB measurements after the laboratory exposures. However, pairwise comparisons indicated that there were no differences between the treatment concentrations (43, 210, and 1100 mg/L) and the control (*p* = 0.498, 0.138, and 0.144, respectively; Supporting Information, Figure S5).

The increase in foot movement activity can be interpreted as an escape reaction to the discomfort caused by a high sulfate concentration, and hence, as an indication of stress. It has been suggested that ion balance disruption is the mechanism of sulfate toxicity to aquatic invertebrates (Pond et al., 2008). Signs of more severe stress, such as reduced filtration activity or entire shell closing, were not observed in our tests. The algal consumption measurements showed that the mussels were actively filtrating water at all the sulfate concentrations. However, the decrease in algal density during the feeding experiment was greatest in the control treatment and apparently declined with increasing sulfate concentration. This suggests that the mussels were feeding more with the low rather than the highest sulfate concentrations.

To our knowledge, our study is the first to explore *U. crassus* behavior in laboratory exposures. However, sulfate toxicity has been studied with other mussel species and other mollusks (Wang et al., 2016). In a previous study with sodium sulfate, the estimated acute EC50 value for the biomass growth of the juvenile mussel *Lampsilis abrupta* was 2253 mg/L SO_4_, and the chronic 20% effect concentration (EC20) value was 696 mg/L SO_4_ (Wang et al., 2016). In accordance with our results, Wang et al. (2016) also observed increased foot movement with high sulfate concentrations. These and our results support the conclusion that sulfate is harmful to mussels at very high concentrations. Long‐term exposure may have effects at lower concentrations.

### Benthic algal accrual

**Figure 4:**
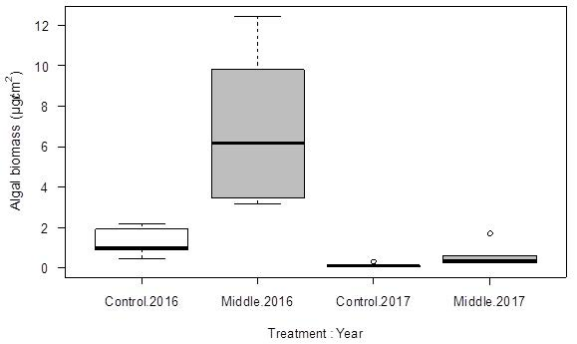
The biomass of benthic algae measured via chlorophyll a (µg cm^−2^) with a spectrophotometer from ceramic tiles incubated for a 7‐week period from August to October in 2016 and 2017 at a reference site above the gypsum treatment area (A1) and in the gypsum spread area (A2).

The measures of the benthic algal biomass accrual through the two methods (using BenthoTorch and a laboratory spectrophotometer) were highly correlated with each other (Pearson's *r* = 0.88; *p* < 0.01). At high biomasses (when the laboratory method indicated more than 3 µg/cm^2^), however, the in situ BenthoTorch measurements indicated lower biomass values than the conventional laboratory method. In the present study we report the results based only on the laboratory biomass estimates (Figure 4), but the results based on the BenthoTorch field probe were qualitatively similar (Supporting Information, Figure S6). The values from laboratory spectrophotometry were likely more accurate estimates for higher biomasses because the BenthoTorch field probe measures only the upper biofilm layer (Echenique‐Subiabre et al., 2016).

There were considerable differences in algal biomass accrual between the sites and years. The algal accrual was higher in the middle reach, A2, than in the upper reach, A1 (*F* [1, 28] = 50; *p* < 0.001), and higher in 2016 than in 2017 (*F* [1, 28] = 145; *p* < 0.001; Figure 4). However, there was no site–year interaction effect (*F* [1, 28] = 0.001; *p* = 0.97) and hence no sign of gypsum treatment effect on benthic algal accrual.

We anticipated that gypsum treatment could either decrease periphyton growth (via decreased dissolved nutrient concentrations) or increase it (via increased light). The study result was expected because the sulfate concentrations remained lower than the previously reported effective concentrations for algae (Elphick et al., 2011) and because gypsum amendment had no apparent effect on the prevailing dissolved phosphorus concentrations or turbidity. Moreover, discerning the effects of many factors, even counteracting ones, on the algal accrual in the field is challenging.

### Mussel abundance

Five mussel species were found from the Savijoki (Supporting Information, Table S1). The *U. crassus* were found only in the lower reaches (sites M14–18), and the total density was also clearly higher there than in the upper reaches. There were no differences in total density (*F* = 0.471; *p* = 0.494) by year, even though in the three sites below and closest to the treatment area, the density seemed to increase consistently after the treatment (Supporting Information, Table S1). The density of the endangered *U. crassus* also did not vary by year (*F* = 0.889; *p* = 0.459) in the lower reaches, where the species was found. Even though the present survey data do not unequivocally show a lack of effect, the apparent effects were positive rather than negative. Signs of positive effects on the mussels can be expected due to the decreased concentration of inorganic suspended solids (Tuttle‐Raycraft et al., 2017), but such a change in water quality was minor in our case.

### Fish assemblages

The fish catch consisted of five species. Stone loach (*Barbatula barbatula*) was found in all the samples and was the only species encountered in the F1 reference site, where it was more abundant than in the other sites. European bullhead (*Cottus gobio*) was also common and relatively abundant both before and after the gypsum treatment, but fewer brown trout (*S. trutta*) were found (Table 3). Chub (*Leuciscus cephalus*) and gudgeon (*Gobio gobio*) were sporadically captured only before the gypsum treatment (Table 3).

**Table 3:** Electrofished area and estimated densities (individuals 100 m^−2^) of fish in the Savijoki River before (B, 2012 or 2013) and after (A, 2017) the gypsum treatment

Site (year)		Area (m^2^)	Total density	*Barbatula barbatula*	*Cottus gobio*	*Gobio gobio*	*Salmo trutta*	*Leuciscus cephalus*
F4	B (2012)	442	7.3	4.3	1.8	0.5	0	0.7
	A (2017)	195	10.3	3.6	6.2	0	0.5	0
F2	B (2013)	210	6.7	3.8	2.4	0.5	0	0
	A (2017)	360	9.2	2.8	6.4	0	0	0
F3 tributary	B (2012)	100	23	6	0	0	17	0
	A (2017)	160	5	4.4	0	0	0.6	0
F1	A (2017)	300	8.3	8.3	0	0	0	0

There were no significant differences in total fish density (paired *t*‐test; *t* = −0.6; *df* = 2; *p* = 0.608) and in the densities of *C. gobio* (*t* = 2.0; *df* = 2; *p* = 0.184) and *S. trutta* (*t* = −0.95; *df* = 2; *p* = 0.441) before and after the gypsum spread, although the density of *C. gobio* tended to be greater after the gypsum treatment (Table 3). The estimated density of *B. barbatula* was smaller after the gypsum treatment (Table 3), but the difference was only borderline significant (*t* = −4.2; *df* = 2; *p* = 0.053). In F3, the estimated density of *S. trutta* was substantially smaller after the gypsum treatment (0.6 individuals/100 m^2^) than before the gypsum treatment (17 individuals/100 m^2^).

The estimated fish densities before and after the gypsum treatment were broadly similar. *Gobio gobio* and *L. cephalus* were absent from the catch after the gypsum treatment, but this could just be by chance because the species were also very few before the treatment. The smaller numbers of *S. trutta* after the gypsum treatment can be related to fisheries management because many more juvenile trout had been stocked into the F3 before the electrofishing in 2012 than in 2017 (Fisheries manager, O. Ylönen, Länsi‐Suomen kalatalouskeskus ry, Finland, personal communication).

Even if we cannot completely rule out the negative effects of gypsum treatment on fish populations, the effects in the Savijoki are likely to be marginal or nonexistent because the average and even peak sulfate concentrations in the Savijoki have been substantially lower than those previously suggested as harmful to freshwater fish (Elphick et al., 2011). However, further experimental studies and longer monitoring are required to arrive at more credible and generalizable conclusions. Due to the high natural annual variation (see Muotka & Syrjänen, 2007), any reliable assessment of the anthropogenic impacts on fish populations will require monitoring of both the impact and reference sites over several years before and after the gypsum treatment (see Louhi et al., 2016).

Fish communities in boreal streams are naturally species poor (Sutela et al., 2010). Moreover, because the Savijoki River catchment is heavily impacted by agricultural diffuse pollution, the fish communities are already impacted by agricultural stressors such as excessive sedimentation and nutrient pollution. Thus, the effects of sulfate can be obscured by the more dominant stressors, especially sedimentation, which is especially harmful for salmonids and other rheophilic fish species such as stone loach and bullhead (Kemp et al., 2011). Fish communities can be more sensitive to sulfate stress in more pristine ecosystems than in agricultural streams.

### Survival and development of trout embryos

Large amounts of fine particles accumulated in the incubation cylinders at all the sites (Supporting Information, Table S4). According to the visual estimate, the mean proportion of sand or smaller fines in the cylinders varied from 33% to 35% between the sites. In sieving, the mean sum of the less than 0.5, 0.5–1, and 1–2 mm size classes was 8%–10% and 2%–4% in the three studied cylinders and baskets, respectively.

The mean embryo survival rate was 78% (range: 62%–88%) in E3 in March, but it was 13% (0–38), 7% (0–32), and 26% (0–76) in E2, E1, and E3 in April, respectively (Supporting Information, Table S4). In the two sampling occasions in May, there were very few individuals alive (0%, 3%, and 1% and 0%, 0%, and 9% in E2, E1, and E3, respectively). All the survivors were unhatched embryos in April but hatched alevins in May. Some dead hatched alevins were observed in the Savijoki cylinders. There were no statistical differences in survival rate between the sites (*F* [2, 22] = 0.7; *p* = 0.49) or between the sampling rounds (*F* [2, 22] = 1.9; *p* = 0.17).

In the experiment, the microenvironment of the baskets (depth, flow rate, and substrate particles; Supporting Information, Tables S2 and S3) corresponded to real trout redds as characterized by Syrjänen et al. (2014). Thus, the constructed hydrological microenvironment probably did not cause high mortality, which was likely due to the substantial sedimentation of fine inorganic material into the baskets and cylinders, caused by high discharge in late Autumn 2017 and early winter 2018 (Supporting Information, Figure S1). The negative effect of the fine particles on the survival rate of the salmonid embryos is well documented (see Chapman, 1988) and is attributable to at least two mechanisms. First, the sedimentation of fines can limit the oxygen availability and/or metabolic waste removal of the embryos (Greig et al., 2005), and second, the sedimentation of fines can form a hard layer preventing the emergence of the hatched embryos to open water (Chapman, 1988; Sternecker & Geist, 2010). Moreover, the unusually thick ice cover in the Savijoki prevented egg sampling in March and thus hampered the experiment.

Despite the aforementioned problems in the present study, the egg cylinders and baskets can be used in forthcoming assessments of gypsum treatment or similar treatments. The method has proved useful in previous studies, in which trout alevins survived well in rivers with good water quality (Syrjänen et al., 2008). However, when any treatment is assessed, the incubation should be implemented at fixed reference and impact sites at least once but preferably both before and after the treatment.

In previous chronic laboratory sulfate exposures, fish embryos were found to be one of the most sensitive life stages. For example, the 21‐day 10% lethal concentration (LC10) for rainbow trout embryos was either 175 or 300 mg/L depending on the hardness of the test water (Sahlin & Ågerstrand, 2018). However, such concentrations were only transiently reached in our study river, suggesting no risks to the reproduction of brown trout.

### Indirect effects of gypsum

The reduced turbidity and phosphorus concentrations caused by gypsum can have a positive effect on the biota. Filtering animals such as mussels can benefit from the reduced inorganic turbidity, hampering their feeding. Reduced sedimentation of fines will also benefit the survival and development of fish embryos. Furthermore, submerged primary producers (algae and macrophytes) will likely benefit from the increased light availability, although this was not observed in the present study.

In our pilot study, the effect of gypsum on the phosphorus load was monitored in two sites along the main river channel, where the effect was diluted by the water from the unamended fields and lands of other use types. Estimating the effect on loads differs from estimating the effect on ecological response. When one is estimating the effect on loads, the use of a flow‐weighted mean concentration results in more correct estimates than the use of arithmetical means due to the positive correlation between concentration and flow. The arithmetical means, however, are more relevant in describing the environment of biota. There were only minimal changes in the arithmetic mean turbidity and phosphorus concentration (Table 2). However, a larger change can be found if the share of amended fields in the total catchment area is substantially larger than in this study (18%).

## CONCLUSIONS

The Finnish government currently aims to amend at least 50 000 hectares of agricultural fields in southwestern Finland with gypsum. Such gypsum treatment can have pronounced potential benefits to the coastal waters. This may also apply to the ecological state of the riverine environment provided that a sufficiently large area of the upstream catchment will be treated. The threats posed by sulfate, however, need to be considered. In our pilot study, gypsum treatment did not raise the sulfate concentrations to a level that is even just close to critical for the biota included in the risk assessment. However, we cannot rule out the possible adverse effects of gypsum treatment on other species based on the ecotoxicity analyses of Sahlin and Ågerstrand (2018) and the precautionary principle. The results of our risk assessment considering both exposure and responses are context specific and do not cover long‐term effects. Therefore, to detect both the possible risks and benefits, we advocate regular monitoring of the river water quality and the biota in the future treatment areas. Inclusion of proper temporal and spatial controls in the monitoring schemes is crucial. In our pilot study, gypsum was spread only in 18% of the total catchment area. In future gypsum amendments, the sulfate effects should be monitored at the sites with the highest anticipated increase in sulfate concentration: in subcatchments with a high share of amended fields in the catchment area.

## Supporting Information

The Supporting information are available on the Wiley Online Library at https://doi.org/10.1002/etc.5248.

## Author Contributions Statement

Jukka Aroviita: Conception and design of experiments; writing; technical and editorial assistance. Petri Ekholm: Conception and design of experiments; writing; technical and editorial assistance. Heikki Hämäläinen: Conception and design of experiments; writing; technical and editorial assistance. Rami Laaksonen: Conception and design of experiments; performance of experiments. Matti T. Leppänen: Conception and design of experiments; writing; technical and editorial editing. Johanna Salmelin: Conception and design of experiments; performance of experiments. Jukka T. Syrjänen: Conception and design of experiments; writing; technical and editorial assistance. Hanna Arola: Performance of experiments. Maija Hannula: Performance of experiments. Tiina Laamanen: Performance of experiments. Krista Rantamo: Performance of experiments; writing; technical and editorial assistance. Jarno Turunen: Performance of experiments. Antti Taskinen: Writing; technical and editorial assistance.

## Supporting information

This article includes online‐only Supporting Information.

Supporting information.

## Data Availability

Data, associated metadata, and calculation tools are in the possession of Finnish Environment Institute and available from the corresponding author (matti.t.leppanen@syke.fi).
