# Supplementary material for: Risk Assessment of Gypsum Amendment on Agricultural Fields: Effects of Sulfate on Riverine Biota
Source: Environ Toxicol Chem. 2021 Dec 29;41(1):108–21. doi: 10.1002/etc.5248 (PMC9302980; doi:10.1002/etc.5248)
Supplement: Supplementary file 1 — Supporting information. [file ETC-41-108-s001.docx]

Supplemental data

**Risk assessment of gypsum amendment on agricultural fields: Effects of sulfate on riverine biota**

The supplemental data file contains details on the water chemistry changes before, during and after the gypsum amendment (Figures S1, S2, S3, S4). Survival of glochidia (S5) and behaviorial activity of mussels at the laboratory gypsum exposure (S6), algal biomass changes at field sites measured with a Benthotorch fluorometer (S7) and mussel densities at the field sites (Table S1). Tables S2-S6 describe the trout embryo incubation results and the exposure conditions. In addition, detailed explanation on the estimation of sulfate concentrations from electrical conductivity is described.


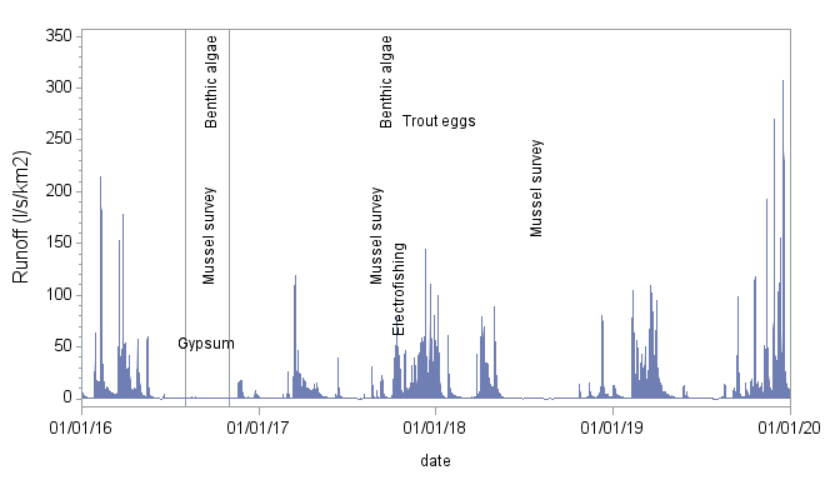


Figure S1. Timing of gypsum amendment (the vertical lines), the field mussel, fish and benthic algal surveys and trout embryo *in situ* exposures, and hourly runoff in the reference (W1 in Figure 1) area in the Savijoki River.

Estimation of sulfate concentration in the Savijoki River

To determine the maximum values of sulfate concentration after the gypsum amendment, two models for the relationship between sulfate concentration and conductivity were applied. The data consisted of sulfate concentrations of the manual water samples and simultaneous electric conductivity measurements of the online sensors at stations W1, W2 and W3. The data was further divided into five periods, the one before the gypsum amendment being about five months and the four ones after gypsum amendment being about a year each. Therefore, the data was separated into 15 station-period combinations in which the number of observations varied between 10 and 27. After fitting the models, their mean predictions and the 95% confidence intervals for sulfate concentration were obtained using all the hourly electric conductivity observations. The prediction data consisted of 3936–10224 observations in each group.

The first model was a nonlinear mixed effects model which was fitted with R version 4.0.2 (R Core Team, 2020) and maximum likelihood method using nlme package (Pinheiro et al. 2020). It can be represented as

${SO4}_{ij}=a_{ij}+{Cond}_{ij}^{b_{ij}}+\varepsilon_{ij}$ (1)

where

*SO4_ij_* = sulfate concentration

*Cond_ij_* = electric conductivity

*a_ij_* , *b_ij_* = parameters

*ε_ij_* = error

*i* = index for observation stations (1–3)

*j* = index for observation periods (1–5)

Parameters *a*_ij_ ja *b*_ij_ consist of both fixed and random effects and can be represented as:

$a_{ij}=c+c_{i}+c_{ij}$ (2)

$b_{ij}=d+d_{i}+d_{ij}$ (3)

where

*c, d* = parameters for fixed effects

*c_i_* , *d_i_* = parameters for random effects of observation stations

*c_ij_* , *d_ij_* = parameters for random effects of observation periods of each station

The mean predictions and the 95% confidence intervals were obtained by nlraa R package with a method using information criterion weights (Miguez 2021).

The second model was a nonlinear model having the same form as Eq. 1 but with fixed effects only fitted separately for each 15 station and period combinations. The model’s least-squares estimates of the parameters were determined with nls package using the Gauss-Newton algorithm. The mean prediction and the 95% confidence interval were calculated by investr R package (Greenwell and Schubert Gabban 2014) based on the linear approximation described in Baty *et al.* (2015).

Here, the results of the first model only are shown (Fig. S2) since it produced slightly higher sulfate concentration predictions. The effect of gypsum amendment can obviously be seen in W2 and W3 in the first period after the amendment, but it levels off soon thereafter. The sulfate concentration stays always below 400 mg/l with 95 % confidence level.


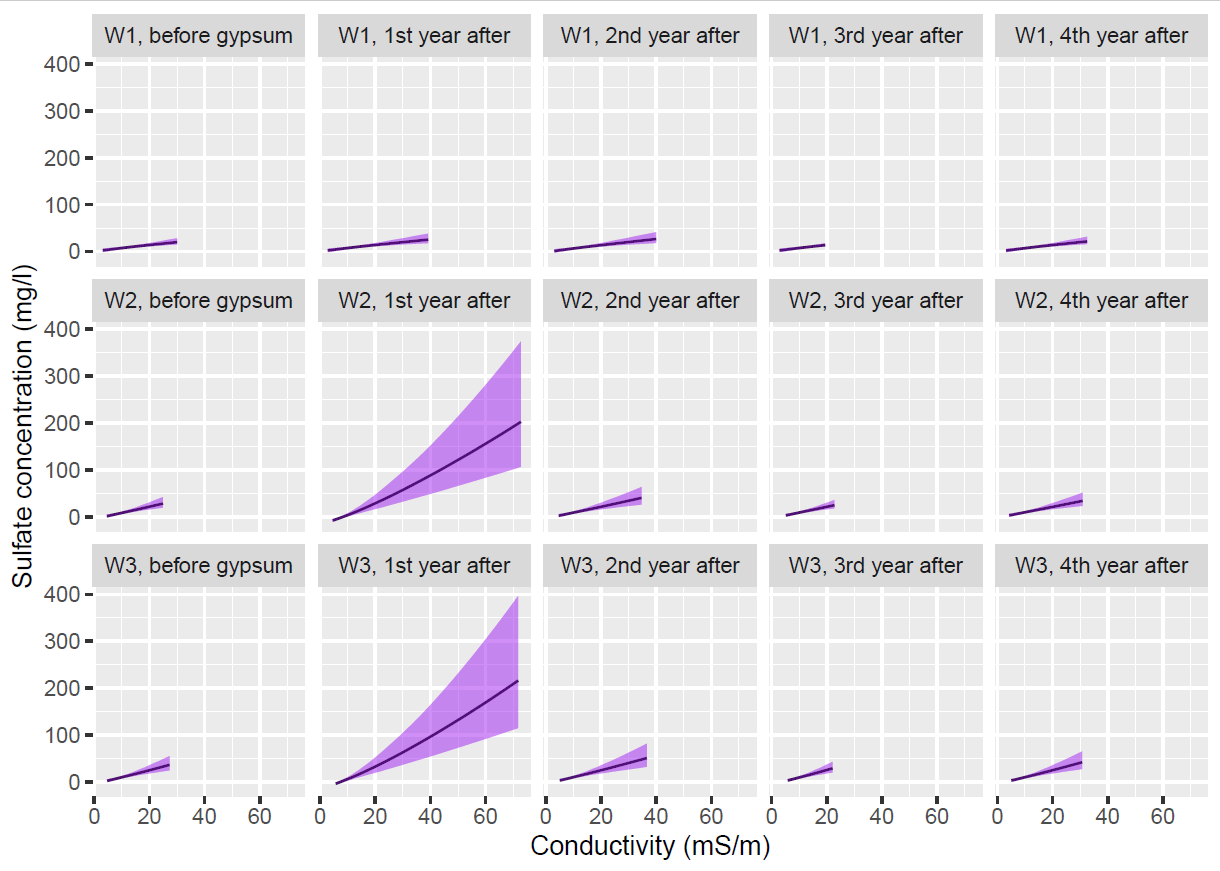


Figure S2. Mean predicted sulfate concentration with 95% confidence interval of the nonlinear mixed effect model.


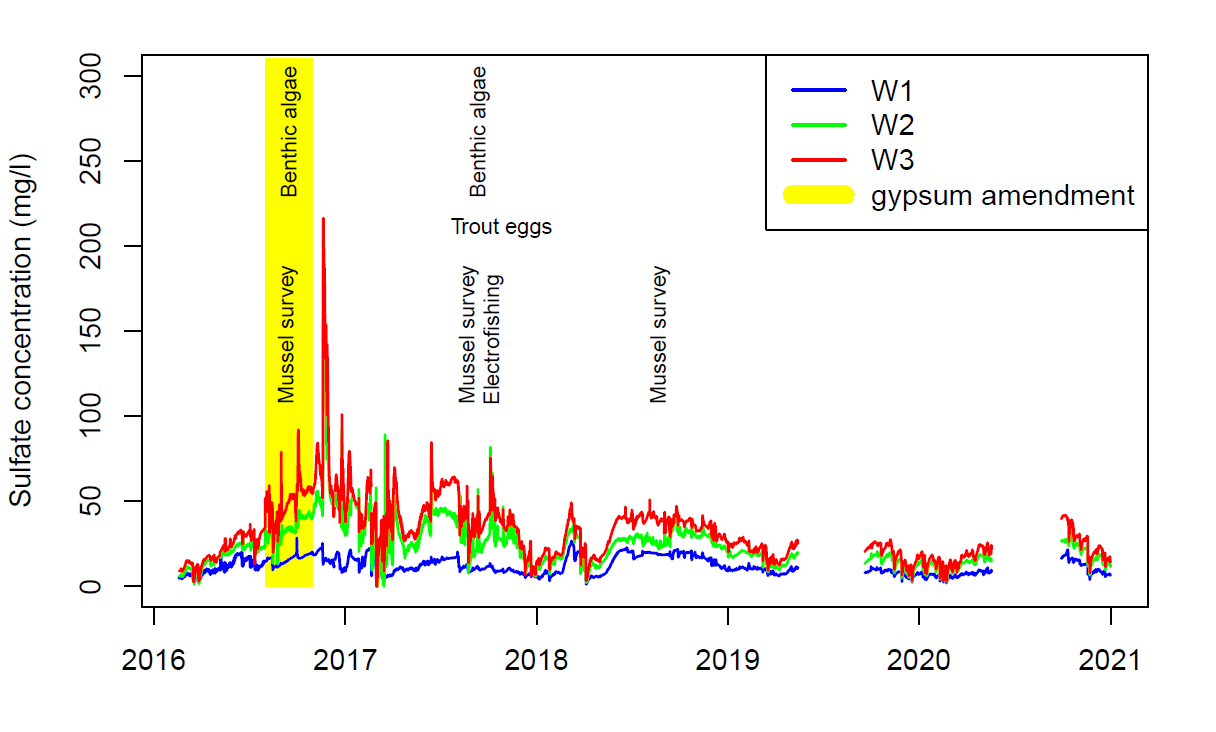


Figure S3. Timing of gypsum amendment (yellow band), the field mussel, fish and benthic algal surveys and trout embryo *in situ* exposures, and the hourly concentration of sulfate in the reference (control) area (W1) and in the upper (W2) and the lower gypsum (W3) area. Sulfate concentrations are estimated based on electric conductivity as described above.

Figure S4. Mean molar concentration (+ standard deviation, SD) of the major ions in the Savijoki River (lower gypsum site, W3) before the gypsum amendment and 0 – 2 months and 3 – 8 months after the amendment.


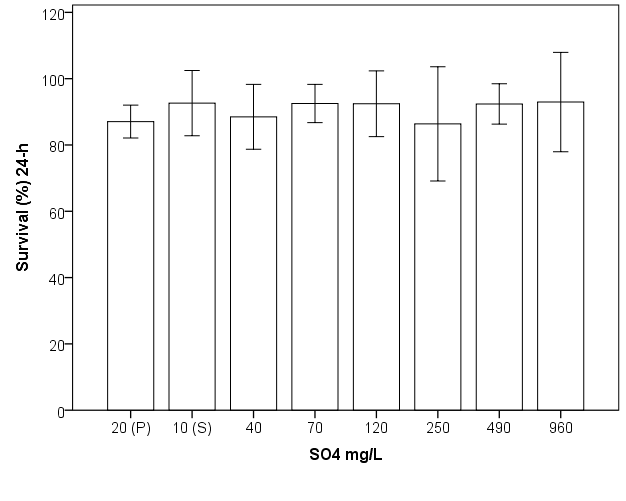

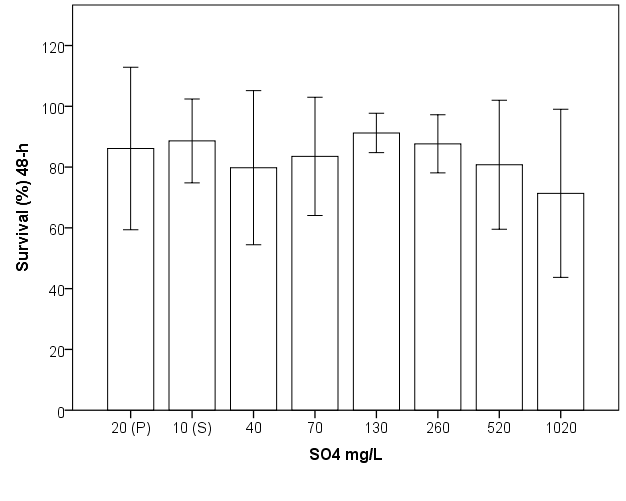


Figure S5. Mean survival ± SD (%) of *U. crassus* glochidia after 24-h and 48-h exposures in the Perniönjoki River control (P), the Savijoki River control (S), and in the exposure concentrations of 40–1020 mg/L SO_4_. Concentrations in the river water controls were 20 mg/L SO_4_ (Perniönjoki) and 10 mg/L SO_4_ (Savijoki).


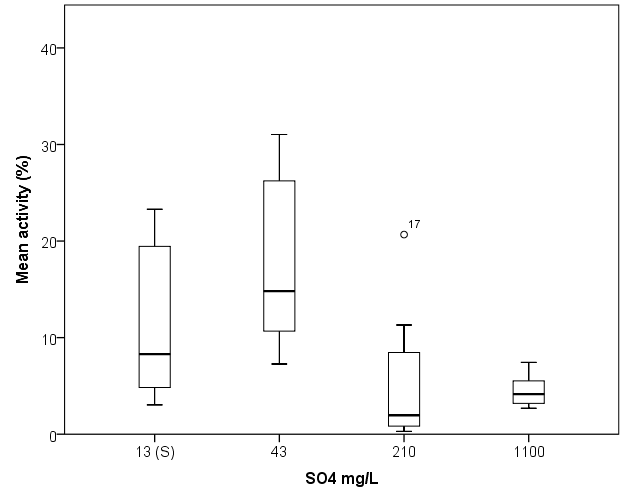


Figure S6. Boxplots (min, 1^st^ quartile, median, 3^rd^ quartile, max) of adult *U. crassus* behavioral activity as mean percentage (%) of time spent in the frequency range of 0.5–2.5 Hz in the control Savijoki River (13 mg/L, S), and in three exposure concentrations. Open circle denotes an outlier (>1.5 times the interquartile range) observation, and the number 17 the respective observation in the data set.

Figure S7. Biomass of benthic algae measured via chlorophyll a (µg/cm^2^) by *in situ* Benthotorch fluorometer from ceramic tiles incubated for a 7-week period from August to October in 2016 and 2017 at a control/reference site (A1, see Figure 1) and in the lower gypsum spreading area (A2).

Table S1. Numbers and densities of different mussel species found and the studied surface areas.

| Site | Year | *Uc* | *Uc* 1/m2 | *Up* | *Up* 1/m2 | *Ut* | *Ut* 1/m2 | *Pc* | *Pc* 1/m2 | *Aa* | *Aa* 1/m2 | Σ | Σ 1/m² | Area m^2^ |
| --- | --- | --- | --- | --- | --- | --- | --- | --- | --- | --- | --- | --- | --- | --- |
| M5 | 2016 |  |  |  |  |  |  | 10 | 0,15 | 1 | 0,01 | 11 | 0,16 | 68 |
|  | 2017 |  |  |  |  | 1 | 0,01 | 14 | 0,21 | 2 | 0,03 | 17 | 0,25 |  |
|  | 2018 |  |  |  |  |  |  | 10 | 0,15 | 1 | 0,01 | 11 | 0,16 |  |
| M11 | 2016 |  |  |  |  |  |  | 23 | 0,31 | 1 | 0,01 | 24 | 0,32 | 75 |
|  | 2017 |  |  |  |  |  |  | 35 | 0,47 | 3 | 0,04 | 38 | 0,51 |  |
|  | 2018 |  |  | 1 | 0,01 |  |  | 68 | 0,91 | 21 | 0,28 | 90 | 1,21 |  |
| M12 | 2016 |  |  | 13 | 0,09 |  |  | 31 | 0,20 |  |  | 44 | 0,29 | 153 |
|  | 2017 |  |  | 22 | 0,14 |  |  | 37 | 0,24 |  |  | 59 | 0,39 |  |
|  | 2018 |  |  | 71 | 0,47 |  |  | 45 | 0,30 | 9 | 0,08 | 125 | 0,82 |  |
| M13 | 2016 |  |  | 1 | 0,01 |  |  | 2 | 0,02 |  |  | 3 | 0,03 | 99 |
|  | 2017 |  |  | 7 | 0,07 |  |  | 5 | 0,05 |  |  | 12 | 0,12 |  |
|  | 2018 |  |  | 19 | 0,19 |  |  | 6 | 0,06 | 1 | 0,01 | 26 | 0,26 |  |
| M14 | 2016 | 3 | 0,17 | 21 | 1,17 | 37 | 2,06 |  |  | 1 | 0,06 | 62 | 3,44 | 18 |
|  | 2017 | 13 | 0,72 | 20 | 1,11 | 55 | 3,06 | 5 | 0,28 |  |  | 93 | 5,17 |  |
|  | 2018 | 9 | 0,50 | 13 | 0,72 | 61 | 3,39 | 1 | 0,06 |  |  | 84 | 4,67 |  |
| M16 | 2016 | 5 | 0,19 | 46 | 1,77 | 37 | 1,42 | 24 | 0,92 |  |  | 112 | 4,31 | 26 |
|  | 2017 | 5 | 0,19 | 28 | 1,08 | 17 | 0,65 | 12 | 0,46 |  |  | 62 | 2,38 |  |
|  | 2018 | 3 | 0,12 | 35 | 1,35 | 46 | 1,77 | 8 | 0,31 |  |  | 92 | 3,54 |  |
| M17 | 2016 | 10 | 0,38 | 41 | 1,58 | 25 | 0,96 | 8 | 0,31 |  |  | 84 | 3,23 | 26 |
|  | 2017 | 8 | 0,31 | 25 | 0,96 | 22 | 0,85 | 6 | 0,23 |  |  | 61 | 2,35 |  |
|  | 2018 | 11 | 0,42 | 39 | 1,50 | 33 | 1,27 | 5 | 0,19 |  |  | 88 | 3,38 |  |
| M18 | 2016 | 11 | 0,31 | 15 | 3,19 | 71 | 2,73 | 12 | 0,46 |  |  | 184 | 7,08 | 26 |
|  | 2017 | 14 | 0,39 | 11 | 0,42 | 33 | 0,92 | 33 | 0,92 |  |  | 92 | 2,56 | 36 |
|  | 2018 | 9 | 0,25 | 8 | 0,31 | 25 | 0,69 | 8 | 0,22 |  |  | 58 | 1,61 |  |

Uc *Unio crassus*, Up *Unio pictorum*, Ut *Unio tumidus*, Pc *Pseudanodonta complanata*, Aa *Anodonta anatina*

Table S2. Water depth (cm) 20 cm in front of the basket (1), on the basket (2 and 3) and 20 cm behind the basket (4) at each field visit and the total means of depths for the whole incubation period. The basket codes Down, Mid and Up indicate the location of the baskets in the streams.

| Site and | | 26 Oct 2017 | | | | | 12 Jan 2018 | | | | 25 Mar 2018 | | | | | 19 Apr 2018 | | | | 8 May 2018 | | | | | 21 May 2018 | | | | Total mean | | | | |
| --- | --- | --- | --- | --- | --- | --- | --- | --- | --- | --- | --- | --- | --- | --- | --- | --- | --- | --- | --- | --- | --- | --- | --- | --- | --- | --- | --- | --- | --- | --- | --- | --- | --- |
| basket | 1 | | 2 | 3 | 4 | 1 | | 2 | 3 | 4 | 1 | 2 | 3 | 4 | 1 | | 2 | 3 | 4 | | 1 | 2 | 3 | 4 | 1 | 2 | 3 | 4 | 1 | 2 | 3 | 4 |  |
| Savijoki, E1 |  | |  |  |  |  | |  |  |  |  |  |  |  |  | |  |  |  | |  |  |  |  |  |  |  |  |  |  |  |  |  |
| Down | 22 | | 25 | 23 | 28 | 22 | | 23 | 23 | 33 | - | - | - | - | 40 | | 34 | 33 | 41 | | 24 | 23 | 24 | 28 | 16 | 7 | 8 | 11 | 25 | 22 | 22 | 28 |  |
| Mid | 35 | | 35 | 34 | 37 | 44 | | 32 | 33 | 43 | - | - | - | - | 51 | | 47 | 48 | 52 | | 43 | 35 | 35 | 39 | 22 | 22 | 22 | 11 | 39 | 34 | 34 | 36 |  |
| Up | 29 | | 23 | 22 | 28 | 33 | | 22 | 22 | 30 | - | - | - | - | 39 | | 34 | 36 | 40 | | 22 | 24 | 25 | 31 | 11 | 15 | 12 | 19 | 27 | 24 | 23 | 30 |  |
| Savijoki, E2 |  | |  |  |  |  | |  |  |  |  |  |  |  |  | |  |  |  | |  |  |  |  |  |  |  |  |  |  |  |  |  |
| Down | 29 | | 25 | 25 | 30 | 50 | | 50 | 47 | 52 | - | - | - | - | 53 | | 43 | 43 | 47 | | 32 | 30 | 28 | 37 | 16 | 11 | 13 | 16 | 36 | 32 | 31 | 36 |  |
| Mid | 31 | | 29 | 30 | 29 | 50 | | 54 | 54 | 57 | - | - | - | - | 45 | | 46 | 46 | 50 | | 32 | 31 | 27 | 32 | 19 | 15 | 17 | 21 | 35 | 35 | 35 | 38 |  |
| Up | 17 | | 19 | 19 | 18 | 46 | | 45 | 42 | 53 | - | - | - | - | 40 | | 38 | 40 | 43 | | 23 | 24 | 20 | 33 | 14 | 7 | 8 | 15 | 28 | 27 | 26 | 32 |  |
| E3, Ext. reference |  | |  |  |  |  | |  |  |  |  |  |  |  |  | |  |  |  | |  |  |  |  |  |  |  |  |  |  |  |  |  |
| Down | 26 | | 29 | 28 | 35 | 40 | | 32 | 31 | 36 | 15 | 8 | 8 | 10 | 43 | | 39 | 37 | 41 | | 37 | 30 | 26 | 36 | 15 | 12 | 14 | 16 | 29 | 25 | 24 | 29 |  |
| Mid | 25 | | 27 | 27 | 26 | 34 | | 32 | 31 | 33 | 6 | 9 | 9 | 10 | 34 | | 36 | 35 | 35 | | 28 | 30 | 26 | 30 | 12 | 11 | 13 | 12 | 23 | 24 | 24 | 24 |  |
| Up | 29 | | 28 | 27 | 33 | 34 | | 32 | 32 | 34 | 2 | 11 | 10 | 15 | 37 | | 36 | 37 | 38 | | 32 | 31 | 31 | 33 | 6 | 16 | 15 | 18 | 23 | 26 | 25 | 29 |  |

Table S3. Flow velocity (cm/s) 20 cm in front of the basket (1), on the basket (2 and 3) and 20 cm behind the basket (4) at 2 cm above the bottom (lower figure) and 60 % depth (upper figure) at each field visit and their total mean. Flow velocities were not measured in the beginning of the incubations (26 Oct 2017) and in March from the Savijoki river sites. The basket codes Down, Mid and Up indicate the location of the baskets in the streams.

| Site and | | 12 Jan 2018 | | | | | 25 Mar 2018 | | | | 19 Apr4 2018 | | | | | 8 May 2018 | | | | 21 May 2018 | | | | | | Total mean | | | |  |
| --- | --- | --- | --- | --- | --- | --- | --- | --- | --- | --- | --- | --- | --- | --- | --- | --- | --- | --- | --- | --- | --- | --- | --- | --- | --- | --- | --- | --- | --- | --- |
| basket | 1 | | 2 | 3 | 4 | 1 | | 2 | 3 | 4 | 1 | 2 | 3 | 4 | 1 | | 2 | 3 | 4 | | 1 | 2 | 3 | 4 | 1 | | 2 | 3 | 4 | |
| Savijoki, E1 |  | |  |  |  |  | |  |  |  |  |  |  |  |  | |  |  |  | |  |  |  |  |  | |  |  |  | |
| Down | 48 | | 53 | 55 | 48 | - | | - | - | - | 60 | 48 | 49 | 42 | 43 | | 37 | 34 | 33 | | 3 | 5 | 7 | 3 | 39 | | 36 | 36 | 32 | |
|  | 25 | | 39 | 26 | 12 |  | |  |  |  | 26 | 11 | 29 | 5 | 19 | | 27 | 12 | 2 | | 0 | 1 | 2 | 0 | 18 | | 20 | 17 | 5 | |
| Mid | 14 | | 25 | 18 | 9 | - | | - | - | - | 32 | 38 | 28 | 29 | 16 | | 14 | 16 | 9 | | 3 | 3 | 1 | 3 | 16 | | 20 | 16 | 13 | |
|  | 0 | | 9 | 6 | 5 |  | |  |  |  | 3 | 8 | 5 | 5 | 2 | | 3 | 2 | 2 | | 1 | 0 | 0 | 1 | 2 | | 5 | 3 | 3 | |
| Up | 54 | | 63 | 64 | 49 | - | | - | - | - | 47 | 51 | 58 | 60 | 21 | | 23 | 31 | 28 | | 5 | 4 | 6 | 7 | 32 | | 35 | 40 | 36 | |
|  | 13 | | 42 | 10 | 0 |  | |  |  |  | 13 | 8 | 10 | 4 | 12 | | 9 | 11 | 1 | | 0 | 0 | 0 | 1 | 10 | | 15 | 8 | 2 | |
| Savijoki, E2 |  | |  |  |  |  | |  |  |  |  |  |  |  |  | |  |  |  | |  |  |  |  |  | |  |  |  | |
| Down | 80 | | 91 | 96 | 95 | - | | - | - | - | 13 | 65 | 70 | 77 | 44 | | 47 | 51 | 46 | | 1 | 10 | 12 | 6 | 35 | | 53 | 57 | 56 | |
|  | 27 | | 20 | 44 | 23 |  | |  |  |  | 5 | 10 | 8 | 14 | 24 | | 3 | 10 | 5 | | 5 | 6 | 3 | 0 | 15 | | 10 | 16 | 11 | |
| Mid | 80 | | 77 | 90 | 90 | - | | - | - | - | 24 | 41 | 27 | 48 | 26 | | 41 | 38 | 47 | | 11 | 28 | 33 | 29 | 35 | | 47 | 47 | 54 | |
|  | 35 | | 7 | 47 | 7 |  | |  |  |  | 13 | 4 | 5 | 5 | 4 | | 34 | 44 | 10 | | 2 | 0 | 0 | 8 | 14 | | 11 | 24 | 8 | |
| Up | 80 | | 70 | 71 | 70 | - | | - | - | - | 85 | 71 | 62 | 68 | 60 | | 66 | 63 | 55 | | 39 | 46 | 46 | 9 | 66 | | 63 | 61 | 51 | |
|  | 7 | | 50 | 51 | 10 |  | |  |  |  | 9 | 15 | 7 | 8 | 45 | | 21 | 54 | 11 | | 28 | 10 | 1 | 4 | 22 | | 24 | 28 | 8 | |
| E3, ext.,  reference |  | |  |  |  |  | |  |  |  |  |  |  |  |  | |  |  |  | |  |  |  |  |  | |  |  |  | |
| Down | 78 | | 89 | 89 | 1 | 16 | | 15 | 13 | 3 | 38 | 65 | 58 | 50 | 29 | | 52 | 62 | 38 | | 7 | 32 | 28 | 8 | 34 | | 51 | 50 | 20 | |
|  | 15 | | 17 | 29 | 20 | 17 | | 7 | 6 | 1 | 7 | 2 | 5 | 3 | 6 | | 19 | 32 | 1 | | 2 | 19 | 6 | 3 | 9 | | 13 | 16 | 6 | |
| Mid | 61 | | 76 | 89 | 97 | 2 | | 1 | 2 | 6 | 42 | 34 | 36 | 32 | 44 | | 32 | 48 | 34 | | 21 | 8 | 8 | 9 | 34 | | 30 | 37 | 36 | |
|  | 29 | | 24 | 32 | 55 | 12 | | 1 | 0 | 6 | 16 | 2 | 3 | 9 | 12 | | 2 | 9 | 11 | | 19 | 1 | 1 | 5 | 18 | | 6 | 9 | 17 | |
| Up | 88 | | 85 | 81 | 81 | * | | 3 | 2 | 7 | 25 | 26 | 22 | 20 | 51 | | 41 | 50 | 53 | | 42 | 36 | 34 | 22 | 52 | | 38 | 38 | 37 | |
|  | 28 | | 25 | 28 | 26 | 15 | | 5 | 1 | 2 | 12 | 7 | 7 | 14 | 2 | | 1 | 9 | 4 | | 46 | 6 | 28 | 2 | 21 | | 9 | 15 | 10 | |

Table S4. Survival and hatching success as proportions from the original egg number and mean total length of the sea trout early life stages in each cylinder at each sampling day at 2018. Particle accumulation is the visually assessed fraction of fine particles in relation to the total content of the cylinder. Degree days are calculated from fertilization (25 Oct 2017) to each sampling day. The mean embryo length was 14.4 to14.8 mm between the sites on 19 April and 18.6 to 19.0 mm on 8 May. The basket codes Down, Mid and Up indicate the location of the baskets in the streams.

|  | | Survival (%) | | | | | Hatched (%) | | | | | Total length (mm) | | | | Particle accumulation (%) | | | | | | Degree days (°C) | | | |
| --- | --- | --- | --- | --- | --- | --- | --- | --- | --- | --- | --- | --- | --- | --- | --- | --- | --- | --- | --- | --- | --- | --- | --- | --- | --- |
| Site and basket | 25 Mar | | 19 Apr | 8 May | 21 May | 25 Mar | | 19 Apr | 8 May | 21 May | 25 Mar | | 19 Apr | 8 May | 21 May | 25 Mar | 19 Apr | 8 May | | 21 May | | 25 Mar | 19 Apr | 8 May | 21 May |
| Savijoki, E1 |  | |  |  |  |  | |  |  |  |  | |  |  |  |  |  |  | |  | |  |  |  |  |
| Down | - | | 22 | 10 | 0 | - | | 0 | 14 | 0 | - | | 14.4 | 19.0 | - | - | 17 | 33 | 33 | | 222 | | 241 | 350 | 522 |
| Mid | - | | 0 | 0 | 0 | - | | 0 | 0 | 0 | - | | - | - | - | - | 50 | 33 | 33 | | 222 | | 241 | 350 | 522 |
| Up | - | | 0 | 0 | 0 | - | | 0 | 0 | 0 | - | | - | - | - | - | 33 | 33 | 33 | | 222 | | 241 | 350 | 522 |
| Savijoki, E2 |  | |  |  |  |  | |  |  |  |  | |  |  |  |  |  |  |  | |  | |  |  |  |
| Down | - | | 0 | 0 | 0 | - | | 0 | 0 | 0 | - | | - | - | - | - | 33 | 13 | 40 | | 219 | | 238 | 358 | 528 |
| Mid | - | | 0 | 0 | 0 | - | | 0 | 4 | 2 | - | | - | - | - | - | 40 | 33 | 40 | | 219 | | 238 | 358 | 528 |
| Up | - | | 38 | 0 | 0 | - | | 0 | 2 | 0 | - | | 14.8 | - | - | - | 33 | 40 | 40 | | 219 | | 238 | 358 | 528 |
| E3, ext.,  reference |  | |  |  |  |  | |  |  |  |  | |  |  |  |  |  |  |  | |  | |  |  |  |
| Down | 88 | | 76 | 2 | 0 | 0 | | 0 | 2 | 0 | 12.8 | | 14.8 | 18.6 | - | 33 | 25 | 33 | 33 | | 150 | | 177 | 308 | 520 |
| Mid | 84 | | 2 | 0 | 0 | 0 | | 0 | 0 | 0 | 12.1 | | * | - | - | 25 | 25 | 33 | 33 | | 150 | | 177 | 308 | 520 |
| Up | 62 | | 0 | 0 | 26 | 0 | | 0 | 0 | 26 | 12.4 | | - | - | 25.9 | 33 | 50 | 33 | 50 | | 150 | | 177 | 308 | 520 |
| * Embryo broke off during dechorionation. | | | | | | | | | | | | | | | | | | | | | | | | | |

Table S5. Water quality characteristics in the Savijoki River. The mean ± SE, and minimum and maximum in parenthesis, at the reference (W1 in Figure 1) and gypsum sites (W2, W3) during the incubation period. Data obtained from the Finnish Environment Institute Water Quality Database (6/2018, http://www.syke.fi/fi-FI/Avoin_tieto/Ymparistotietojarjestelmat).

| Period and site | Runoff (l/s/km^2^) | | Suspended solids  (mg/l) | Electric conductivity (mS/m) | | pH | Alkalinity (mmol/l) | | SO_4_ (mg/l) | Ca (mg/l) |  |
| --- | --- | --- | --- | --- | --- | --- | --- | --- | --- | --- | --- |
| 26 Oct 2017-19 Apr 2018 |  | |  |  |  | |  |  | |  | |
| Ref.W1 | 16.5 ± 0.3 (0.1–144.2) | | 96.0 ± 1.4 (24.8–660.2) | 14.4 ± 0.1 (3.1–40.0) | 7.1 (6.8–7.5) | | 1.14± 0.37 (0.33–2.70) | 9.6 ± 0.1 (5.0–19.8) | | 10.2 ± 0.1 (3.0–26.4) | |
| W2 |  | | 94.3 ± 1.4 (21.2–519.0) | 17.6 ± 0.1 (4.7–34.6) | 7.3 (7.1–7.7) | | 0.89 ± 0.16 (0.37–1.60) | 24.2 ± 0.2 (10.5–72.3) | | 13.8 ± 0.1 (4.2–32.2) | |
| W3 |  | | 85.5 ± 1.2 (19.4–524.4) | 18.2 ± 0.1 (5.1–35.5) | 7.3 (7.1–7.5) | | 0.93 ± 0.18 (0.40–1.90) | 25.4 ± 0.2 (10.5–76.1) | | 14.4 ± 0.1 (4.4–33.4) | |
| 20 Apr-8 May 2018 | |  |  |  |  | |  |  | |  | |
| Ref. W1 | 17.3 ± 0.6 (8.6–88.6) | | 75.8 ± 2.5 (41.8–591.4) | 9.2 ± 0.0 (8.2–10.5) | 7.2 | | 0.50 | 7.5 ± 0.0 (7.1–8.0) | | 6.9 ± 0.0 (6.2–7.7) | |
| W2 |  | | 89.7 ± 2.5 (52.5–395.7) | 12.9 ± 0.0 (11.4–14.9) | 7.3 | | 0.56 | 15.4 ± 0.1 (13.7–17.8) | | 9.6 ± 0.0 (8.5–11.2) | |
| W3 |  | | 88.7 ± 2.6 (48.9–455.5) | 14.4 ± 0.0 (12.9–16.0) | 7.3 | | 0.58 | 17.2 ± 0.1 (15.3–19.5) | | 10.8 ± 0.0 (9.6–12.1) | |
| 9 May-21 May 2018 | |  |  |  |  | |  |  | |  | |
| Ref. W1 | 3.5 ± 0.1 (0.9–8.8) | | 67.5 ± 0.4 (1.5–88.2) | 13.5 ± 0.1 (10.5–17.9) | 7.5 | | 0.80 | 9.2 ± 0.1 (8.0–11.0) | | 9.6 ± 0.1 (7.7–12.4) | |
| W2 |  | | 66.2 ± 0.7 (1.5–140.1) | 17.4 ± 0.1 (14.9–20.2) | 7.4 | | 0.93 | 21.9 ± 0.2 (17.8–27.3) | | 13.3 ± 0.1 (11.2–15.8) | |
| W3 |  | | 57.9 ± 0.5 (41.8–84.7) | 19.0 ± 0.1 (15.9–22.5) | 6.9 | | 0.76 | 25.0 ± 0.2 (19.3–32.5) | | 14.7 ± 0.1 (12.0–18.1) | |

Table S6. Water quality characteristics in the Järvijoki River (external reference E3 in Figure 1) and the Lake Savojärvi at depth of 0.1–0.5 m in years 2000–2016. The mean ± SE, and minimum and maximum in parenthesis. Data obtained from the Finnish Environment Institute Water Quality Database (6/2018, http://www.syke.fi/fi-FI/Avoin_tieto/Ymparistotietojarjestelmat).

| Period and site | Turbidity (FNU) | pH | Alkalinity (mmol/l) | SO_4_ (mg/l) | Electric conductivity (mS/m) | | Oxygen saturation (%) | | Oxygen concn. (mg/l) |
| --- | --- | --- | --- | --- | --- | --- | --- | --- | --- |
| Järvijoki 2006–2016 | 91 ± 22 (15–540) | 7.0 (6.4–7.7) | 0.68 ± 0.07 (0.36–1.20) | - | | 11 ± 1 (5–24) | 85 ± 2 (73–100) | 10.1 ± 0.5 (7.7–12.9) | |
| Savojärvi 2000–2015 | 5 ± 0.9 (1.4–13) | 5.9 (4.9–6.6) | 0.11 ± 0.02 (0.04–0.34) | 3.6 | | 4 ± 0 (3–10) | 76 ± 5 (8–96) | 8.4 ± 0.5 (1.1–11.5) | |
|  |  |  |  |  | |  |  |  | |

REFERENCES

Baty F, Ritz C, Charles S, Brutsche M, Flandrois J-P, Delignette-Muller M-L. 2015. Toolbox for Nonlinear Regression in R: The Package nlstools. Journal of Statistical Software, 66(5), 1-21. URL: http://www.jstatsoft.org/v66/i05/.

Greenwell B. M. and Schubert Kabban C. M. 2014. investr: An R Package for Inverse Estimation. The R Journal, 6(1), 90-100. URL: http://journal.r-project.org/archive/2014-1/greenwell-kabban.pdf

Miguez F. 2021. nlraa: Nonlinear Regression for Agricultural Applications. R package version 0.89. URL: https://CRAN.R-project.org/package=nlraa

Pinheiro J, Bates D, DebRoy S, Sarkar D, R Core Team 2020. nlme: Linear and Nonlinear Mixed Effects Models. R package version 3.1-148. URL: https://CRAN.R-project.org/package=nlme

R Core Team 2020. R: A language and environment for statistical computing. R Foundation for Statistical Computing, Vienna, Austria. URL https://www.R-project.org/
